# Tuberous Sclerosis Complex With Multiple Organ Tumors: Case Report and Literature Review

**DOI:** 10.3389/fonc.2022.916016

**Published:** 2022-07-19

**Authors:** Xinhe Zhang, Xinping Zhong, Xuyong Lin, Xuedan Li, Haoyu Tian, Bing Chang, Ying Wang, Jing Tong, Ningning Wang, Dan Li, Xiuli Jin, Die Huang, Yanmeng Wang, Huipeng Cui, Lin Guan, Yiling Li

**Affiliations:** ^1^ Gastroenterology Department, The First Affiliated Hospital of China Medical University, Shenyang, China; ^2^ Department of Hepatobiliary Surgery, The First Affiliated Hospital of China Medical University, Shenyang, China; ^3^ Department of Pathology, The First Affiliated Hospital of China Medical University, Shenyang, China; ^4^ Radiology Department, The First Affiliated Hospital of China Medical University, Shenyang, China; ^5^ The 3rd Clinical Department, China Medical University, Shenyang, China

**Keywords:** tuberous sclerosis complex, liver perivascular epithelioid tumors, pancreatic neuroendocrine neoplasms, splenic hamartoma, renal angiomyolipoma

## Abstract

Pancreatic neuroendocrine neoplasms (PNEN) are tumors that originate from neuroendocrine cells. Only about 1% patients are related to mutation of tuberous sclerosis complex gene. Here, we reported a rare case with involvement of multiple organs and space-occupying lesions. Initially, the patient was thought to have metastasis of a pancreatic tumor. However, the patient was diagnosed as pancreatic neuroendocrine tumors, liver perivascular epithelioid tumors, splenic hamartoma, and renal angiomyolipoma by pathological examination after surgery. We performed genetic mutation detection to identify that tuberous sclerosis complex 2 gene presented with a heterozygous variant. Tuberous sclerosis often presents with widespread tumors, but it is less common to present with pancreatic neuroendocrine tumors and liver perivascular tumors as highlighted in the case. So we analyzed the relationship between TSC gene mutations and related tumors. And we also reviewed the current molecular mechanisms and treatments for tuberous sclerosis complex.

## Introduction

Neuroendocrine tumors are tumors that originate from neuroendocrine cells and can be distributed throughout the body. The gastrointestinal tract and the pancreas are the most common locations for these tumors (1). The incidence of pancreatic neuroendocrine neoplasms (PNEN) is approximately 5 cases per 100,000, and the rate in women tends to be moderately higher than those in men. PNEN account for 3% of primary pancreatic tumors. Patients present different clinical symptoms according to the endocrine hormones produced by tumors (2). But the variable and atypical clinical manifestations of PNEN patients means that missed and misdiagnoses are relatively common.

Tuberous sclerosis complex (TSC) is an autosomal dominant chronic disease, occurring as a consequence of mutations in the TSC1 gene on chromosome 9q34 or the TSC2 gene on chromosome 16p13. This is a clinically rare condition, with a reported incidence of between 1/6000 and 1/10000 (3). It can cause multi-organ tumors due to mutations in the TSC gene such as the brain, skin, heart, lung, and kidneys. However, accumulation in the pancreas is relatively rare, especially PNEN.

About 10% of PNEN patients are hereditary, mainly related to autosomal dominant inheritance, such as multiple endocrine neoplasms type I (MEN1), neurofibromatosis type I (NF-1), tuberous sclerosis (TSC), etc. MEN1 is the most common type, while TSC type only accounts for about 1%. Here, we reported a patient with multiple organ occupying lesions. The patient was diagnosed with PNEN, renal angiomyolipoma, splenic hamartoma, and liver vascular peripheral epithelioid tumors with the help of surgery and pathological tests. The mutation in the TSC2 gene was found by genetic mutation detection.

## Case report

**Figure 1:**
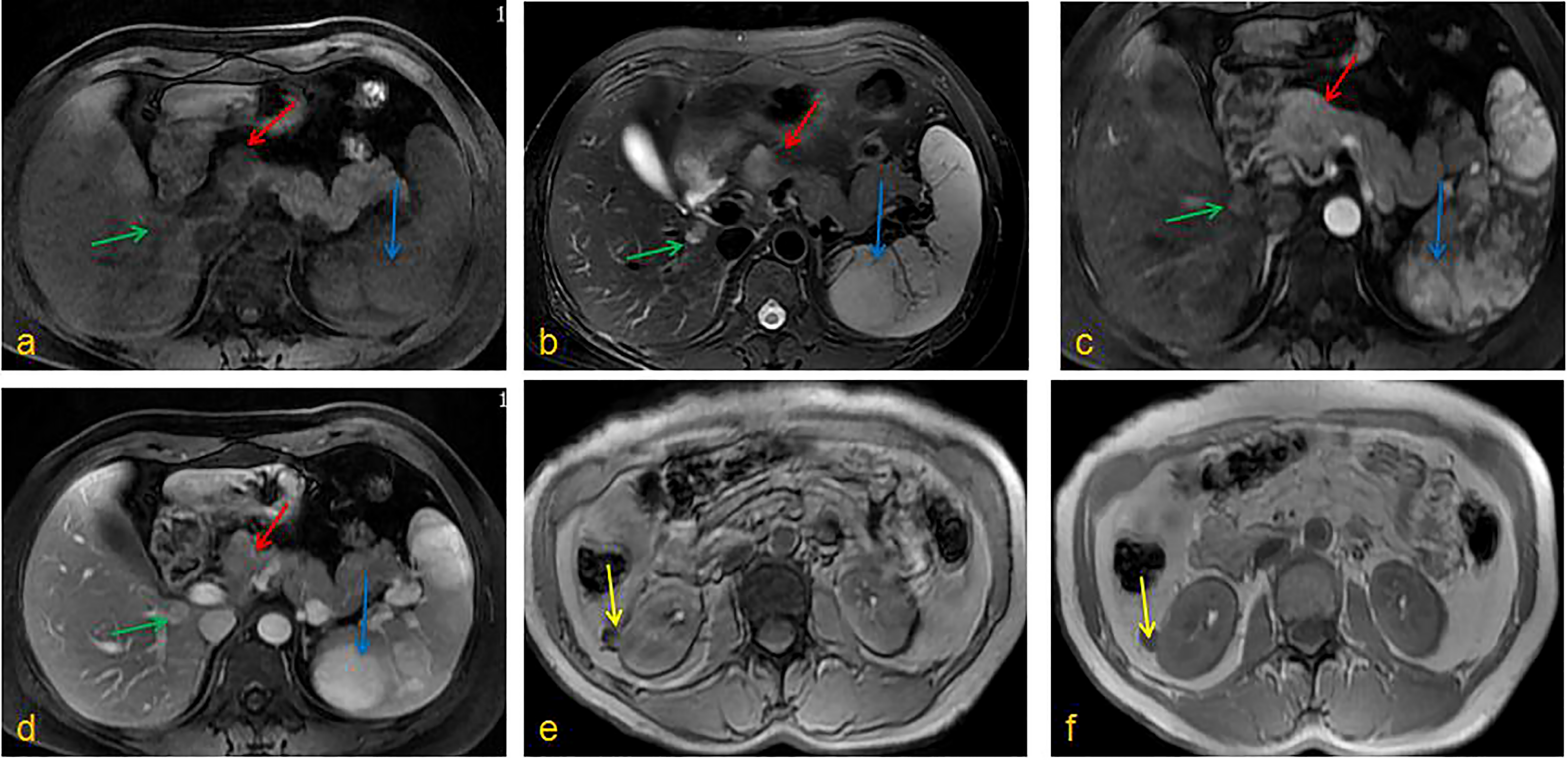
Enhanced MRI of the abdomen. **(A)** T1WI, **(B)** T2WI, **(C)** Enhancement phase, **(D)** Delay phase: the pancreatic head could be seen a clear outline of about 2.5cm in diameter with long T1T2 signal, the lower segment of the right liver had oval shadow of about 1.4cm in diameter with long T1T2 signal, the spleen showed multiple irregular T1 signals and long T2 signals; **(E)** T1WI, **(F)** T2WI, kidneys could be seen round T1 signal and low T2 signal foci, and the enhanced arterial phase showed obvious enhancement. (red arrow: pancreatic tumor; blue arrow: splenic tumor; green arrow: liver tumor; yellow arrow: kidney tumor).

A 43-year-old woman presented to the First Affiliated Hospital of China Medical University with left back pain. However, there was no jaundice, abdominal pain, weight loss, or other symptoms. After admission, relevant laboratory examinations were performed and all within the normal range, including routine blood test, liver function, renal function, coangulation test, immynoglobulin levels, insulin and glucagon, tumor indicators (CEA, AFP, CA125, CA199), etc. Enhanced CT examination of the upper abdomen revealed multiple low-density shadows in the lower right lobe of the liver, head of the pancreas, and spleen. Soft tissue-density nodules were on the surface of the renal cortex. The largest nodule was located in the right kidney and protruded out of the kidney. The arterial phase of the enhanced scan was obviously or unevenly strengthened in the nodules when compared with the surrounding tissue. Enhanced MRI of the abdomen confirmed multiple space-occupying changes in the liver, spleen, pancreas, and kidneys (**Figure 1**). PET-CT further showed that the tumor within the pancreas was malignant and exhibited obvious uptake of fludeoxyglucose (FDG). Both the kidney and spleen were considered more likely to contain benign lesions given that there was no obvious FDG uptake.

**Figure 2:**
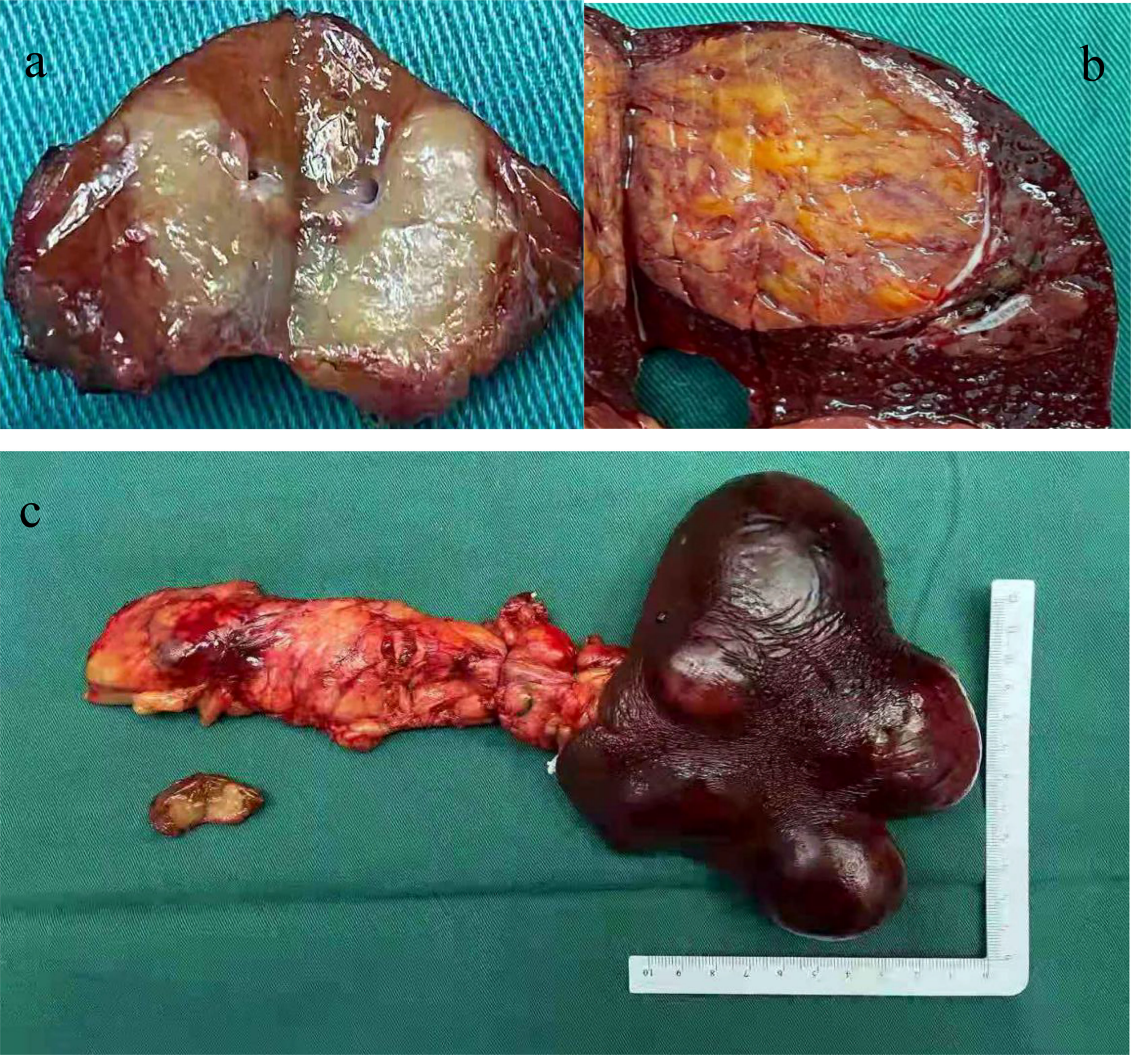
Tumor specimens of surgical excision. **(A)** A tumor with a diameter of about 1 cm was seen in the caudate lobe of the liver with a gray-white nodule in the center; **(B)** The cut surface of the splenic tumor was grayish-red, gray-brown and medium in quality; **(C)** The pancreatic tumor was located on the body of the pancreas with a diameter of about 2.5 cm. The cut surface was grayish yellow and soft with bleeding.

Based on these examination results, the patient underwent surgical resection. After opening the abdomen, a tumor of diameter 2.5 cm was found in the body of the pancreas (**Figure 2**). The tails of the pancreas and spleen were removed. The cut surface of the pancreatic mass was grayish-yellow and soft with hemorrhage. The cut surface of the splenic mass was grayish-red and brown. A tumor with a diameter of approximately 1 cm was observed in the caudate lobe of the liver and presented with gray-white nodules in the center. This tumor was excised in a wedge shape.

**Figure 3:**
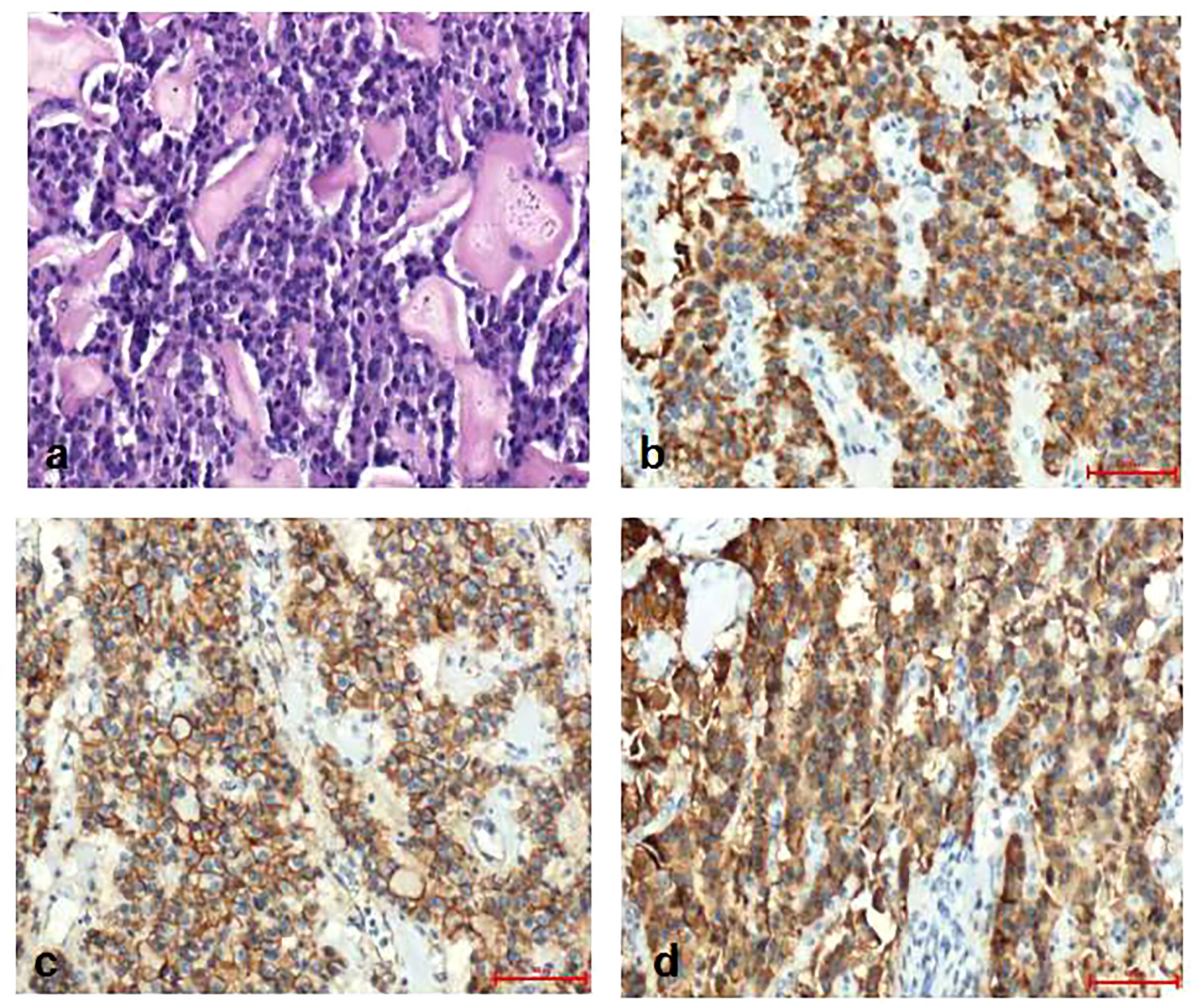
Pathology result of the pancreas. **(A)** Pancreatic tumor cells were mostly distributed in strips or diffuse sheets, with the same cell size; **(B)** Immunohistochemistry CK(PAN)(+); **(C)** Immunohistochemistry SY(+); **(D)** Immunohistochemistry beta -Catenin (cell membrane+).

**Figure 4:**
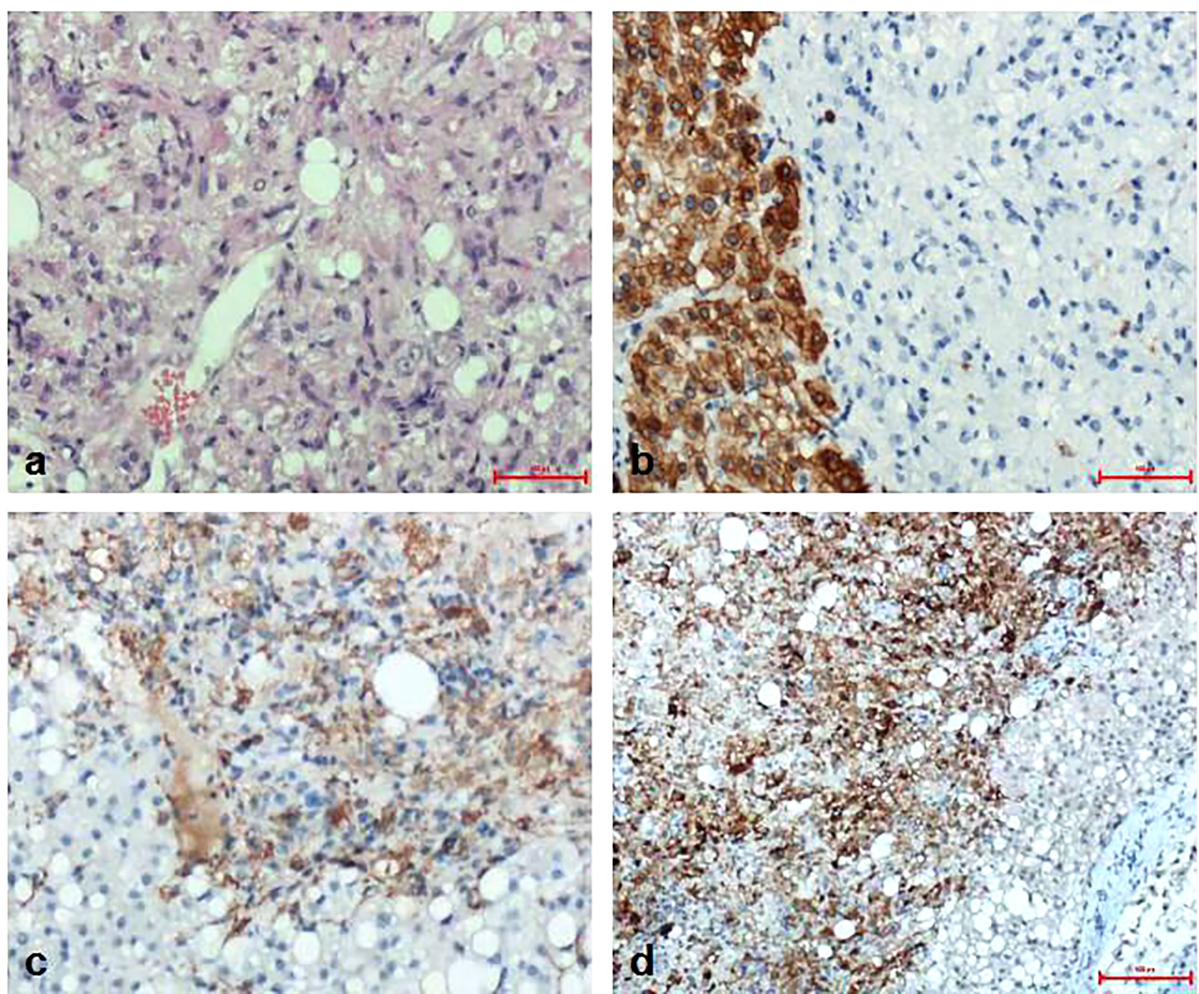
Pathology result of the liver. **(A)** The boundary between the liver tumor and the surrounding liver tissue was unclear. The tumor was the mixture of epithelioid cells, perivascular muscle-like cells and adipocytes in different proportions; **(B)** Immunohistochemistry CK (hepatocyte+); **(C)** Immunohistochemistry Melan A(+); **(D)** Immunohistochemistry SMA (part+).

**Figure 5:**
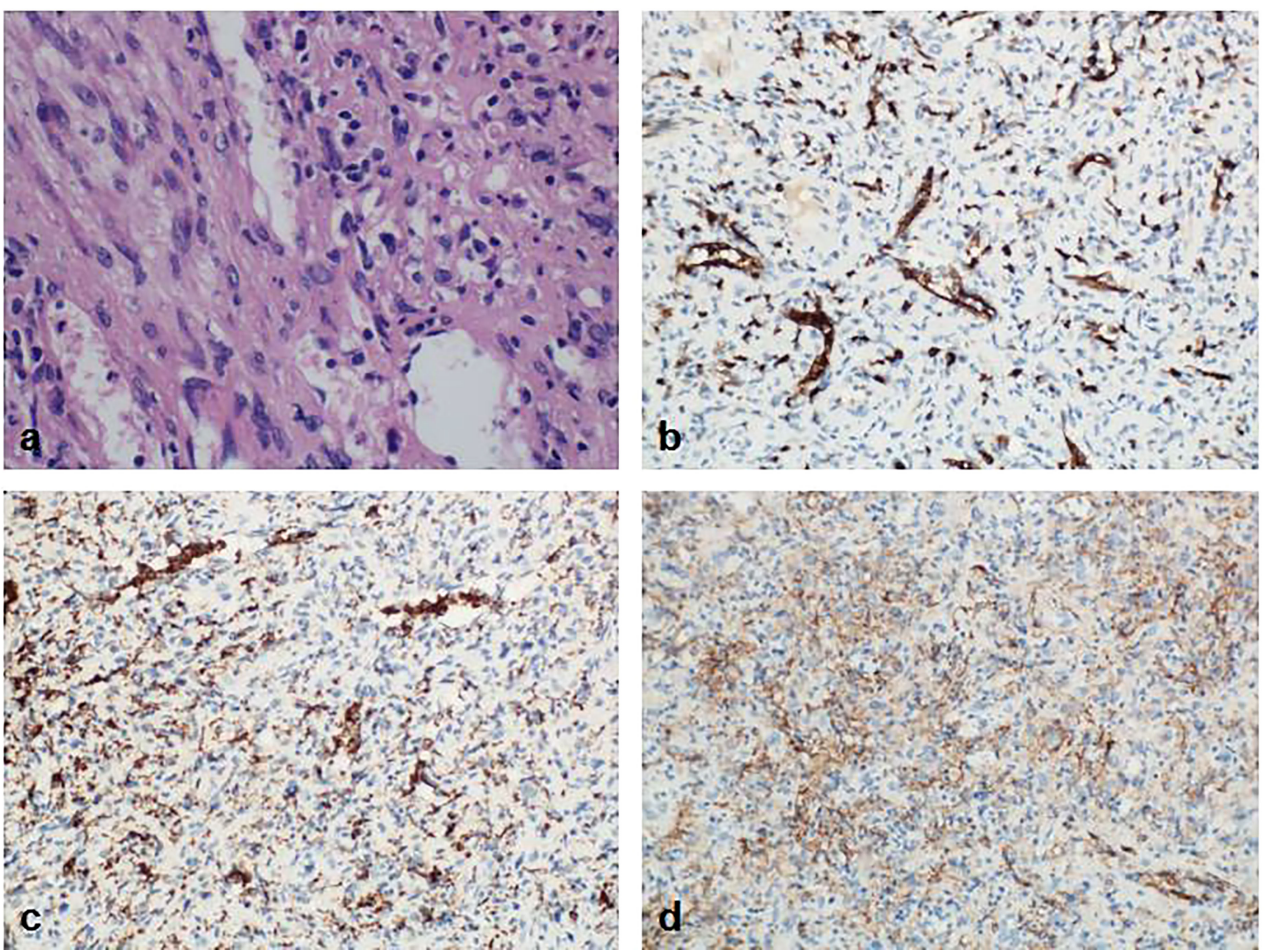
Pathology result of the spleen. **(A)** The splenic tumor was in the shape of a round nodule, with a clear boundary with the surrounding normal spleen tissue. The tumor lost the structure of the red and white pulp of the normal spleen under the microscope. The cells were mainly composed of oval tissue-like cells and spindle-shaped muscle-like cells. Blood vessels were abundant; **(B)** Immunohistochemistry CD8(+); **(C)** Immunohistochemistry CD68(+); **(D)** Immunohistochemistry SMA (interstitial +).

Pathological diagnosis of the spleen, liver, and pancreas was performed during and after the operation. Intraoperative cryopathology suggested that the splenic tumors were likely to be benign lesions, and the tumors in the pancreas were likely to be pancreatic pseudopapillary tumors. Microscopic examination of the paraffin-embedded histopathological section showed that the tumor cells of the pancreas (**Figure 3**) were mostly distributed in strips or diffuse sheets, with the same cell size and characteristic salt and pepper-like nuclei. Immunohistochemistry confirmed that cytokeratin (CK) and beta-catenin were positive, which supported the diagnosis of pancreatic neuroendocrine tumors. The tumor and the surrounding tissues of the liver were unclear (**Figure 4**). The liver-associated tumor was found to contain epithelioid cells, perivascular muscle-like cells, and adipocytes. Immunohistochemistry revealed that phosphatidylinositol proteoglycan-3 (GPC-3) was negative and Melan A was positive, supporting the diagnosis of a perivascular epithelioid cell tumor (PEcoma). The splenic tumor was a round-shaped nodule (**Figure 5**) with clear boundary. The tumor comprised of uniformly sized oval-shaped tissue-like cells and spindle-shaped muscle-like cells. Immunohistochemistry showed positive staining for both smooth muscle actin (SMA) and cluster of differentiation 68 (CD68), supporting the diagnosis of splenic hamartoma (myoid hamartoma).

**Figure 6:**
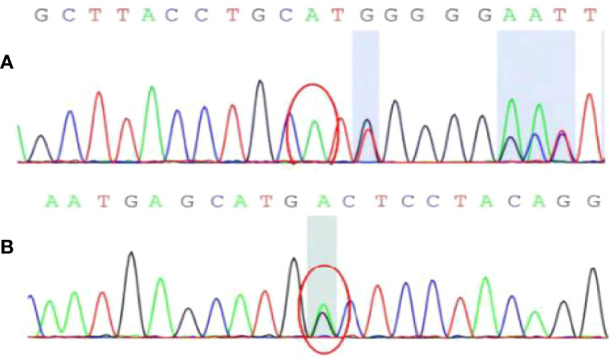
The result of genetic mutation detection. **(A)** BAP1 gene c.1111dupA site with insertion mutation; **(B)** TSC2 gene c.4700G>A (guanine>adenine) site with heterozygous mutation.

**Figure 7:**
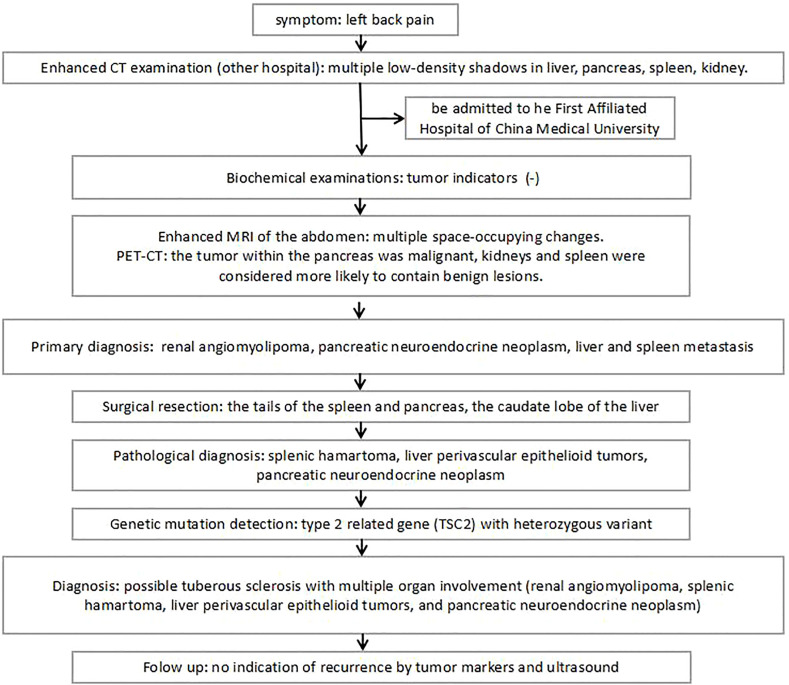
Diagnosis and treatment algorithm based on this case report.

To ascertain the cause of the multiple tumors, we performed genetic mutation detection using the patient’s genomic DNA, which was recovered from the peripheral blood. The results showed that the tumor susceptibility syndrome-related genes BRCA1 related protein 1 (*BAP1*) and tuberous sclerosis complex 2 gene (*TSC2)* each presented with a heterozygous variant (**Figure 6**). These findings are in line with the genetic diagnosis of tuberous sclerosis. In terms of clinical diagnosis, the liver and kidney angiomyolipomas are major indicators, and the splenic hamartoma belongs to non-renal hamartoma. Consequently (4), the most likely diagnosis was tuberous sclerosis with multiple organ involvement. After diagnosis, we completed the examination and did not find the TSC pathological features of other organs (skin and lungs). Tumor markers and ultrasound were re-examined at 3 and 6 months after surgery, and there was no indication of recurrence. The patient’s diagnosis and treatment processes are shown in **Figure 7**.

## Discussion

Initially, the patient was thought to have metastasis of pancreatic endocrine tumor. However, after pathological findings and genetic mutation, the patient was diagnosed as tuberous sclerosis complex.

Aberrations of the TSC genes are associated with a variety of tumors. Skin damage is the most common manifestation, observed in over 95% of cases. Patients may present depigmented spots on the extremities or red hard papules on the face, which then manifest as depigmented spots, facial fibers, and perithyroid fibroma (5). Brain tissue lesions are the main cause of morbidity in children with TSC (6). Specific lesions include cortical nodules, subependymal nodules, and subependymal giant cell astrocytoma, out of which cortical nodules, the most common form of organic brain tissue lesion in TSC patients, is observed in over 88% of children with TSC (7). In addition, TSC involving the heart and lungs mainly manifests as cardiac rhabdomyomas and pulmonary lymphangiomyomas, which can be found in more than 80% of children with TSC (8).

The *TSC* genes are tumor suppressors and produce hamartin and tuberin proteins, which are known to be associated with a variety of tumors. TSC variant identified by sequencing is a known pathogenic variant. Mutations of TSC1 or TSC2 lead to constitutive activation of the small G protein Rheb and the mammalian target of rapamycin (mTOR) pathway, which regulates cell growth and proliferation (9). Associated to this pathway, insulin-like growth factor can cause phosphorylation of AKT and activate PI3K. In turn, activated PI3K converts PIP2 to PIP3 and recruits PDK1 and Akt, thereby activating mTOR (10). Complex of mTOR and Rheb promote the phosphorylation of downstream molecules, consequently promoting protein synthesis. TSC protein is a key regulator of this signaling pathway, which can transform the form of Rheb-GTP to Rheb-GDP, which inhibits the formation of complexes and slows down cell growth (11). Deletion of the *TSC* gene leads to dysregulation of mTOR, which is the regulator of the PI3K/Akt/mTOR signaling pathway. Their activation makes cell cycle regulation out of control and causes tumor progression. Therefore, when the *TSC* gene is mutated, the structure and function of the TSC1/TSC2 complex is abnormal. So the inhibitory effect on mTOR is weakened, resulting in growth of the tumors (12). The pathogenesis of some TSC-related benign tumors requires not only the mutation and inactivation of the *TSC* gene, but also the mutation and inactivation of their alleles, which is the “second hit” (13). These circumstances generate tumors such as facial angiofibroma, renal angiomyolipoma, renal cell carcinoma, and lymphangiomyomatosis.

What we note as unusual is that PNEN is not in the diagnostic criteria for multi-system-involved TSC. This is because most PNEN are closely related to gene such as *ATRX/DAXX*, *MEN-1*, and *MUTYH.* Only a few cases reported that PNEN and TSC are concurrent. Larson (14) retrospectively analyzed the results of 219 TSC patients, of whom 6 patients were previously diagnosed with PNEN through pathological examination. And TSC2 mutation was found in all patients. According to previous reports (15), 37% of pancreatic endocrine tumors have lost the 16p chromosome arm. This is also closely related to the PI3K-Akt-mTOR pathway. Akt activation was observed in PNEN patients. Akt/mTOR is involved in the growth and apoptosis of pancreatic β-cells. Activated mTOR can regulate downstream factors p70S6K and 4EBP-1, and affect the cell growth cycle.

Reports of TSC involving the spleen are rare, especially for splenic hamartoma. An early TSC case was reported in an infant using CT to identify the presence of bilateral renal angiomyolipomas. The splenic mass increased significantly during the 62-month follow-up period. Pathological examination after splenectomy indicated that the tumor was splenic hamartoma. Ultimately, whether it is affected by the mTOR pathway requires further verification.

**Figure 8:**
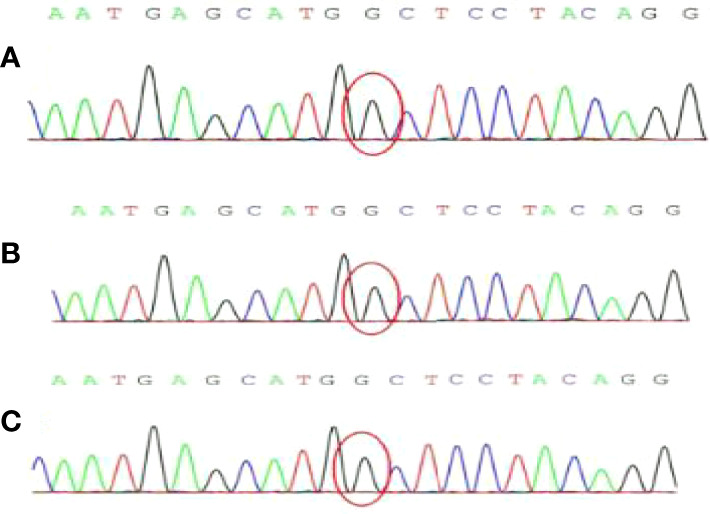
The result of genetic first-generation sequencing detection. No TSC gene mutation was found. **(A)** Hepatocellular carcinoma; **(B)** Cholangiocarcinoma; **(C)** Neuroendocrine tumors.

In addition, PEcoma is related to TSC gene mutations (16). Specifically, 10% to 50% of patients possess TSC2 gene deletion (17). Kenerson (18) confirmed that the high expression of mTOR activity markers and the low expression of Akt in PEcoma are associated with the destruction of TSC function. Pan collected 10 sporadic and two tuberous sclerosis-related PEcomas. The phosphorylation curve shows that the mTOR pathway is activated by the disrupted function of the TSC1/2 complex, which proves that the carcinogenic lineage of PEComa is a unique TSC2-related tumor (19). Therefore, the two-hit mutation of TSC1 or TSC2 is causally associated with the onset of TSC-PEComas. The main disease which must be distinguished from liver PEComa is primary liver cancer. Both of them can show the form of “fast in and fast out” on enhanced CT scans. However, patients with hepatocellular carcinoma usually have a history of liver disease such as hepatitis B and cirrhosis, and AFP level is usually elevated (20). The differences are mainly due to pathology and immunohistochemistry assessment, and the mutant genes of these two liver tumors are different. Most PEcoma patients exhibit mutations in TSC. To further verify the difference, we assessed patients from the First Hospital of China Medical University whose gender or age was similar to this case study and whose conditions included hepatocellular carcinoma, cholangiocarcinoma, and neuroendocrine tumors of the liver. First-generation sequencing of cancer tissue specimens revealed no TSC gene mutations (**Figure 8**). Therefore, genetic testing is a good tool for distinguishing between primary liver cancer and PEcoma, which is difficult to distinguish by other methods. Other diseases that need to be differentiated include liver adenocarcinoma, liver hemangioma, and liver focal nodular hyperplasia, amongst others (21).

Since TSC is a systemic disease, and treatments vary in accordance with the individual’s specific manifestations. Surgical resection is the most important treatment for PEcoma (22). It has been reported that the systematic use of sirolimus, an mTOR inhibitor, can shrink tumors and facilitate surgical resection (23). There are also reports of radiofrequency ablation, chemotherapy, and other methods. But the long-term efficacy of these methods is still uncertain. In addition, because mTOR overexpression plays an important role in the development of PEcoma, mTOR inhibitors may be beneficial for PEcoma treatment. It has been reported that a 17-year-old PEcoma patient had significant reduction in tumor size after 3.5 years of sirolimus treatment (24). Based on the regulatory role of the mTOR pathways, mTORC1 inhibitors support the possibility of treating TSC patients based on the physiological pathogenesis, including renal angiomyolipoma, giant cell subependymal astrocytoma, and lymphatic vessel leiomyomatosis (25). The first generation of mTOR inhibitors, rapamycin and its derivative everolimus, can bind to FK506-binding protein-12 and form a complex, thereby inhibiting mTOR activity. Its effectiveness and safety have been confirmed clinically. A randomized, double-blind, placebo-controlled, international multi-center Phase III clinical trial study showed that 42% (33/79) of the everolimus group had reduced tumor size. The average duration of this reduction was greater than 5 months, while placebo group exhibited no reduction in tumor size (0/39) (26). The FDA approved everolimus for the treatment of TSC-RAML that does not require immediate surgery. Currently, this is the only drug that can be used to treat TSC-RAML.

## Conclusion

The onset of tuberous sclerosis is rare. The genetic cause is mutations in the *TSC1* and *TSC2* genes, which involves the mTOR signaling pathway and lead to multiple system involvement. The clinical manifestations and types of disease are highly variable. Among them, the skin and nervous system are the most involved, causing skin nodules and epilepsy, but this condition can also rarely involve organs such as the liver, gallbladder, and spleen, causing rare symptoms and forming PEcoma or neuroendocrine tumors. Therefore, timely genetic testing should be considered when multiple organs and multiple types of space-occupying lesions occur. The accurate diagnosis of TSC disease is based on genetic testing and clinical manifestations, as proposed by the International TSC Consensus Group. Treatments should focus on symptom management, and some forms of the disease can be treated with mTOR inhibitors.

## Author Contributions

Writing-Original Draft Preparation, XHZ; Writing-Review and Editing, YL; Operation and case, XPZ; Image, Xd.L; Pathology, Xy.L; Gene detection, XJ; Data collection, LG, NW, HT, DL, BCYMW, JT, DH, YW, and HC. All authors contributed to the article and approved the submitted version.

## Conflict of Interest

The authors declare that the research was conducted in the absence of any commercial or financial relationships that could be construed as a potential conflict of interest.

## Publisher’s Note

All claims expressed in this article are solely those of the authors and do not necessarily represent those of their affiliated organizations, or those of the publisher, the editors and the reviewers. Any product that may be evaluated in this article, or claim that may be made by its manufacturer, is not guaranteed or endorsed by the publisher.
